# Action Video Game Training for Healthy Adults: A Meta-Analytic Study

**DOI:** 10.3389/fpsyg.2016.00907

**Published:** 2016-06-17

**Authors:** Ping Wang, Han-Hui Liu, Xing-Ting Zhu, Tian Meng, Hui-Jie Li, Xi-Nian Zuo

**Affiliations:** ^1^Key Laboratory of Behavioral Science, Institute of Psychology, Chinese Academy of SciencesBeijing, China; ^2^University of Chinese Academy of SciencesBeijing, China; ^3^Youth Work Department, Chinese Youth University for Political StudiesBeijing, China

**Keywords:** action video game training, healthy adults, meta-analysis, cognitive functions, moderator variable

## Abstract

Action video game (AVG) has attracted increasing attention from both the public and from researchers. More and more studies found video game training improved a variety of cognitive functions. However, it remains controversial whether healthy adults can benefit from AVG training, and whether young and older adults benefit similarly from AVG training. In the present study, we aimed to quantitatively assess the AVG training effect on the cognitive ability of adults and to compare the training effects on young and older adults by conducting a meta-analysis on previous findings. We systematically searched video game training studies published between January 1986 and July 2015. Twenty studies were included in the present meta-analysis, for a total of 313 participants included in the training group and 323 participants in the control group. The results demonstrate that healthy adults achieve moderate benefit from AVG training in overall cognitive ability and moderate to small benefit in specific cognitive domains. In contrast, young adults gain more benefits from AVG training than older adults in both overall cognition and specific cognitive domains. Age, education, and some methodological factors, such as the session duration, session number, total training duration, and control group type, modulated the training effects. These meta-analytic findings provide evidence that AVG training may serve as an efficient way to improve the cognitive performance of healthy adults. We also discussed several directions for future AVG training studies.

## Introduction

The video game industry has developed for more than 40 years since the release of the first arcade machine and household gaming console. Originally, video games were usually associated with aggressive behaviors, poor school performance, video game addiction, motion sickness, seizures, and instability of mood (e.g., Kasteleijn-Nolst Trenite et al., 2002; Gentile et al., 2004; Polman et al., 2008). Gradually, researchers found that video games improved a variety of cognitive abilities, including spatial visualization, decision-making, visuospatial, and language processing (e.g., Dorval and Pepin, 1986; Ball et al., 2002; Castel et al., 2005; Bialystok, 2006).

Action video game (AVG), one of the genres of video game, refers to games that have fast motion, require vigilant monitoring of the visual periphery, and need to track multiple targets simultaneously (Green and Bavelier, 2003). Due to multiple cognitive domains being involved in AVG, AVG has gradually attracted many researchers’ interest and has become one of the main intervention types to enhance cognitive functions in healthy adults. Previous studies found participants in different ages benefited from AVG training. Green and Bavelier (2003, 2006a,b, 2007) conducted a series of AVG training studies on young adults and reported that AVG training brought positive impacts on various aspects of cognition functions, such as visual divided attention, visuospatial processing, working memory, and processing speed. Oei and Patterson (2013) used the AVG game *Modern Combat* to train 16 young adults for 4 weeks (20 h), they found that the participants improved their cognitive control and multiple object tracking ability. Furthermore, researchers also found that young AVG players improved the attentional control (Chisholm and Kingstone, 2015) and executive function (Hutchinson et al., 2015). Several studies reported that older adults also benefited from AVG training. Basak et al. (2008) revealed that older adults in the AVG training group presented significant improvements in working memory, abstract reasoning, distractor inhibition, and mental rotation and a significant reduction in task-switching costs relative to older adults in control group. McDermott (2013) found that older adults increased their attention control ability in multiple areas after 50 h of AVG training and this effect last for 3–4 months. Other studies also showed that prolonged experience of playing AVG produced positive cognitive effects on older adults (Stern et al., 2011; Maillot et al., 2012).

However, previous AVG training studies produced mixed and even controversial conclusions. For example, two studies reported task switch cost reductions after 15 (Strobach et al., 2012) and 50 h (Green et al., 2012) of AVG training, whereas Boot et al. (2008) failed to find enhancements in task switching performance following 21.5 h AVG training. Two previous training studies investigated whether AVG training improved visual search ability in non-video-game players. Wu and Spence (2013) demonstrated greater accuracy and shorter search time in their AVG training group, whereas Oei and Patterson (2013) did not find improvement in visual search in training group participants. Furthermore, the sample sizes in most previous training studies were very small, which may affect the reliability and validity of the AVG training effect. Although several excellent reviews and meta-analyses have investigated the effect of video game training on cognition (Powers et al., 2013; Lampit et al., 2014; Toril et al., 2014), these meta-analyses did not focus on AVG and did not compare the training effect on young and older adults.

In the present meta-analysis, we aimed to systematically examine the extent and nature of AVG training on cognitive functions in healthy adults. In particular, we investigated and compared AVG training effects in young and older adults. In the present analyses, effect sizes were computed through the comparison of pre- and post-cognitive measures of both the AVG group and control group.

## Materials and Methods

### Literature Search

To identify pertinent articles, a two-staged literature search was carried out. First, an online Web of Sciences, PubMed, EBSCO, and PsycNET (PsycINFO, PsycARTICLES) database search was performed between January 1986 and July 2015. The search terms were “video game,” “video games,” “videogame,” “videogames,” “video-game,” “computer game,” and “computer games” with different combinations of “intervention,” “training,” and “stimulation.” Second, the reference lists of included articles and several AVG training review papers were scrutinized for studies not indexed in the electronic databases.

### Selection Criteria

#### Inclusion Criteria

The studies were included if they met the following criteria: (i) the participants are healthy adults, and the mean ages are equal to or more than 18 years old; (ii) the training group receives the AVG training; (iii) the study includes a control group; (iv) the study reports the pre- and post-test of the same cognitive outcomes for both the training group and the control group; and (v) the study should provide means, standard deviations (SDs), *t*-test or *F*-test, and *n* values or other data that can be calculated for effect size.

#### Exclusion Criteria

The studies were excluded if: (i) the participants involved in the study are a clinical population or the mean age is less than 18 years old; (ii) the training group does not receive the AVG training; (iii) there is no control group; (iv) the study does not report cognitive outcomes; and (v) the study does not present enough data to calculate effect sizes.

### Data Collection

According to the inclusion and exclusion criteria, two of the authors (PW and TM) judged whether the searched studies should be included in the present meta-analysis. Furthermore, these two authors extracted the demographic variables and cognitive outcomes of the included studies independently. The differences were solved through discussion. When a study included two or more training groups, each AVG training group was considered as a single study (Stern et al., 2011). We contacted the authors to obtain the raw summary data if we could not extract the data from the study reports.

### Outcome Measures

The effects of AVG training were evaluated using a variety of tests. According to previous studies (Li H. et al., 2011; Baniqued et al., 2014), we divided the cognitive functions into processing speed/attention, visuospatial processing, memory, and executive function. Each task in the studies was assigned to only one of these four cognitive domains. Processing speed/attention mainly included digit symbol test, Trail Making Test – Part A, processing speed test, filter task, enumeration task, useful field of vision task, visual search task, and pattern comparison task. Visuospatial ability, which generally refers to skills in representing, transforming, generating, and recalling symbolic, and non-linguistic information (Linn and Petersen, 1985; Halpern, 2000), included mental rotation tests, spatial span test, block design task, center identification task, and crowding thresholds task. Only a small number of studies reported memory tasks and mainly focused on episodic memory and semantic memory tests. Executive function, an umbrella term for functions such as planning, working memory, reasoning, inhibition, mental flexibility as well as monitoring of action (Chan et al., 2008), primarily included working memory, stopping task, Trail Making Test – Part B, Stroop or Stroop-like tasks, flanker task, and Raven’s Advanced Progressive test.

### Data Analysis

We used the Comprehensive Meta-Analysis software (version 2.2; Borenstein et al., 2005) to calculate the effect sizes (Cohen’s *d*; Cohen, 1988) in each study. For most studies, the means and SDs of the pre- and post-test for both the training group and the control group were used to calculate the effect size. The *P*-values, *t*-values, or *F*-values between groups were used if the means and SDs were unavailable. If the individual study provided more than one cognitive test for a certain domain, these tests were combined into a single effect size. Based on Cohen’s *d*, an effect size of less than 0.2 is considered negligible, a value between 0.2 and 0.5 is considered small, a value between 0.5 and 0.8 is considered moderate and a value equal to or greater than 0.8 is considered large (Cohen, 1988). A combined effect size with corresponding confidence intervals (95%) was also presented. To identify the heterogeneity of the included studies, we calculated the heterogeneity statistic *Q* (Lipsey and Wilson, 2000). The Egger’s regression intercept test, as an index of funnel plot, was used to test the publication bias (Egger et al., 1997).

To evaluate the AVG training effect, we first analyzed the overall effect size and separate cognitive domains in healthy adults for all included studies. Effect size was calculated by the standardized mean differences in the pre-test and post-test between the AVG training group and control group. We then performed meta-analyses and calculated effect sizes for overall and specific cognitive functions in both young and older adults. Meta-regression analyses were conducted to identify the potential effects of moderator variables on AVG training.

### Moderator Variables

A series of studies have suggested that the efficacy of video game training or cognitive training may be affected by a number of potential factors. Age was found to be positively correlated with training effects among older adults (Powers et al., 2013; Toril et al., 2014). Meta-analyses showed that there was no effect of the time spent in cognitive training (Karbach and Verhaeghen, 2014; Karr et al., 2014), while other reviews or meta-analyses suggested that the entire training duration, session duration, and number of sessions were negatively correlated with video game training effects (Primack et al., 2012; Lampit et al., 2014). Years of education were considered to be a protective factor for age-related cognitive decline (Anstey et al., 2003). The effects of gender in the study of video game training are debatable. Powers et al. (2013) showed that males benefited more from information processing than females in video game training, whereas other studies found that females benefited more than males in spatial skills after video game training (Subrahmanyam and Greenfield, 1994; Feng et al., 2007). Boot et al. (2011) considered whether control group types influenced the training effect. They claimed that participants in the training group knew that they were receiving a training intervention, and this knowledge, coupled with the belief that the treatment may lead to improvement compared with participants who received no interventions (Boot et al., 2011), may have had an effect on the results. The study quality was believed to potentially influence the training effects (Hemingway et al., 2010; Karch et al., 2013); therefore, we developed 10 items to evaluate study quality of the included studies. These items included a pre-specified hypothesis, participant selection criteria, sample number (more or less than 15 participants), randomization, baseline differences, the blinding of the participants, the blinding of outcome assessors, dropout rate (more or less than 25%), loss of data explained, and the lack of selective outcome reporting. Each item was calculated as one point. The control group type typically included an active-control group and a passive-control group. Participants in the former group received non-AVG training, while the latter group received no intervention. The non-AVG training refers to game interventions without the definite characteristics of AVG, such as a paper-and-pencil activity, three-dimensional puzzle game training, Tetris (a puzzle game) training, and Sims (a strategy game) training. We first calculated the meta-analyses for the studies classified as passive and active-control types and then compared the differences of effect sizes with the *Q*-test. Therefore, the age, training duration, session duration, number of session, education, gender, study quality, and control types were analyzed as moderator variables in the present meta-analyses.

## Results

### Search Results and Data Availability

**FIGURE 1:**
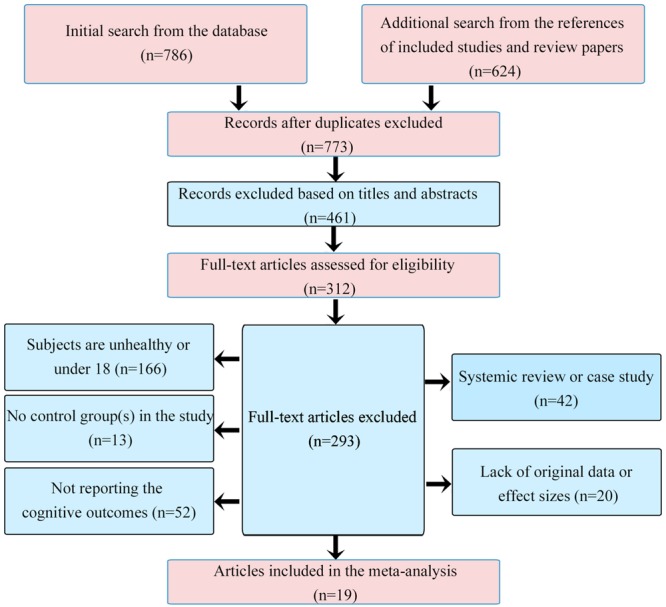
**Flow chart of the study selection process**.

We obtained 786 studies from the online database search and 624 studies from reference searching. After removing the duplicates, we considered 773 studies, of which 754 were excluded for various reasons (see details in **Figure 1**). Nineteen studies fulfilled the inclusion criteria. Because one study included two experimental groups, we divided it into two studies (Stern et al., 2011). Finally, 20 studies were included in the current meta-analysis, for a total of 313 participants included in the training group and 323 participants in the control group. The literature search processes are shown in **Figure 1**. The characteristics of these 20 studies are summarized in **Table 1**.

**Table 1:** Characteristics of included studies in the action video game training meta-analysis.

Author and Year	Experimental group			Control group			Training type		Training time			Outcome measures	Study quality
	*N*	Age	Male ratio (%)	*N*	Age	Male ratio (%)	Experimental group	Control group	SL	SN	TTL		
Basak et al. (2008)	19	69.89	27.8	20	68.88	25	The Rise of Nation	Passive	1.5 h	15	23.5 h	Executive function, attention, visuospatial	8
Belchior et al. (2013)	14	74.8	64.3	13	73.7	53.8	Medal of Honor	Passive	1.5 h	6	9 h	Processing speed	7
Blacker et al. (2014)	17	20.41		17	20.65		Call of Duty	Active			30 h	Executive function, working memory	9
Boot et al. (2013)	14	73	48	20	72	45	Mario Kart DS	Passive	1 h	60	60 h	Processing speed, attention, reasoning, executive function, memory	6
Cherney et al. (2014)	20	20.5	50	20	20.5	50	Wii	Active	0.5 h	2	1 h	Visuospatial	8
Clark et al. (1987)	7	65	57.1	7	74	28.6	Pac Man and Donkey Kong	Passive	7 h+/w	7 w	49 h+	Processing speed	7
Colom et al. (2012)	10	18.95	0	10	18.95	0	Professor Layton and The Pandora’s Box	Passive	4 h/w	4 w	16 h	Executive function, reasoning	7
Green and Bavelier (2006a)	16	21.3	50	16	21	43.75	Unreal Tournament 2004	Active	2 h+/d		30 h	Visuospatial	9
Green and Bavelier (2006b)	9	20.4	44.4	8	19.7	50	Medal of Honor	Active	1 h/d	10 d	10 h	Executive function	8
Green and Bavelier (2007)	16	21.3	50	16	21	43.75	Unreal Tournament 2004	Active	2 h+/d		30 h	Visuospatial	9
Li et al. (2009)	6	26.51	50	7	23.72	71.43	Unreal Tournament 2004	Active	2 h/d		50 h	Visuospatial	8
Li et al. (2010)	14	26	50	11	24.7	63.6	Unreal Tournament 2004	Active	2 h/d		50 h	Attention	8
Maillot et al. (2012)	16	73.47		16	73.47		Nintendo Wii	Passive	1 h	24	24 h	Executive function, processing speed, visuospatial	8
Nelson and Strachan (2009)	10	21.8		16	21		Unreal Tournament 2004	Active	15 min	4	1 h	Processing speed	7
Sanchez (2012)	30		26.7	30		53.3	Combat Evolved	Active			25 min	Visuospatial	8
Schubert et al. (2015)	21	24.7	52.4	21	25.6	47.6	Medal of Honor	Passive	1 h	15	15 h	Executive function, processing speed, working memory, visuospatial	7
Seçer and Satyen (2014)	14	70		15	70		Pac Man	Passive	3 h	3	9 h	Processing speed	6
Stern et al. (2011, study 1)	20	66.34	41	20	66.95	41	Space Fortress (emphasis change)	Passive	1 h	36	36 h	Executive function, visuospatial, memory, attention, language, processing speed	9
Stern et al. (2011, study 2)	20	66.56	50	20	66.95	41	Space Fortress standard instruction	Passive	1 h	36	36 h	Executive function, visuospatial, memory, attention, language, processing speed	9
Wu and Spence (2013)	20	18–25	50	20	18–25	50	First Person Shooter	Active	1–2 h		10 h	Processing speed	8

*Note: SL, session length; SN, session number; TTL, total training length; d, day; h, hour; min, minute; w, week; s, session; +, at least.*

### The AVG Training Effect on Overall Healthy Adults

**FIGURE 2:**
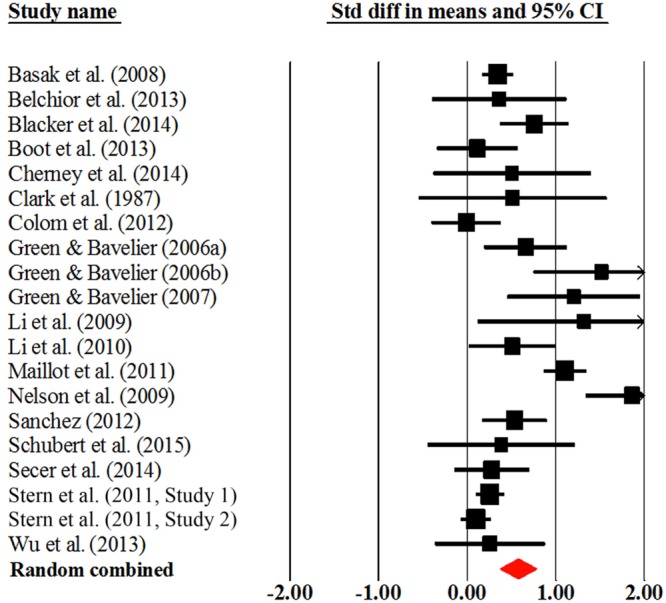
**Overall efficacy of action video game training on all cognitive outcomes in overall adults**.

**Figure 2** presents the forest plot of overall cognitive effects of AVG training for the overall healthy adults. As shown in **Table 2**, the overall effect size was 0.58, showing that AVG training produced moderate effect on overall cognition. The homogeneity test presented significant heterogeneity (*Q* = 103.57, *P* < 0.001), suggesting that the effect sizes across different studies varied significantly.

**Table 2:** The main findings of action video game training on young and older adults.

Cognitive domains	Number of studies	Effect size		Heterogeneity			Egger’s test	
		*d* (SE)	95% CI	Q	*P*	*I*^2^	*t*	*P*
**Young adults**								
Overall cognition	12	0.75 (0.16)	0.43–1.07	42.27	<0.001	73.97	10.00	0.287
Processing speed/ attention	4	0.81 (0.47)	0.11–1.73	19.82	<0.001	84.86	0.30	0.790
Visuospatial ability	6	0.70 (0.12)	0.46–0.94	3.94	0.559	<0.001	2.09	0.105
Executive function	4	0.64 (0.33)	0.01–1.29	14.87	<0.01	79.88	2.00	0.637
**Older adults**								
Overall cognition	8	0.38 (0.13)	0.12–0.64	48.14	<0.001	85.46	0.36	0.728
Processing speed/ attention	8	0.37 (0.20)	-0.02 to 0.76	54.59	<0.001	87.18	0.55	0.601
Memory	3	0.33 (0.19)	-0.03 to 0.71	0.00	0.998	0.00	0.29	0.822
Visuospatial ability	4	0.29 (0.20)	-0.10 to 0.68	7.15	0.067	58.05	0.92	0.455
Executive function	5	0.40 (0.22)	-0.04 to 0.84	14.87	<0.001	88.96	0.32	0.773
**Overall adults**								
Overall cognition	20	0.58 (0.10)	0.37–0.78	103.57	<0.001	81.67	1.84	0.082
Processing speed/ attention	12	0.50 (0.18)	0.14–0.85	81.69	<0.001	86.53	0.15	0.883
Memory	3	0.33 (0.19)	-0.05 to 0.71	<0.001	0.998	<0.001	0.29	0.822
Visuospatial ability	10	0.54 (0.12)	0.30–0.77	15.37	0.081	41.43	0.78	0.458
Executive function	9	0.49 (0.17)	0.15–0.83	51.19	<0.001	84.37	0.13	0.898

*Note: CI, confidence interval; SE, stander error.*

Action video games training had moderate impacts on visuospatial ability (Cohen’s *d* = 0.54) and processing speed/attention (Cohen’s *d* = 0.50). The effect size of executive function was 0.49, approaching moderate magnitude. We also found that overall adults experienced enhancement of a small magnitude in memory function (Cohen’s *d* = 0.33). As shown in **Table 2**, Egger’s test for the overall cognition and specific cognitive domains were not significant, suggesting that there was no publication bias.

### The Effect of AVG Training on Young and Older Adults

**FIGURE 3:**
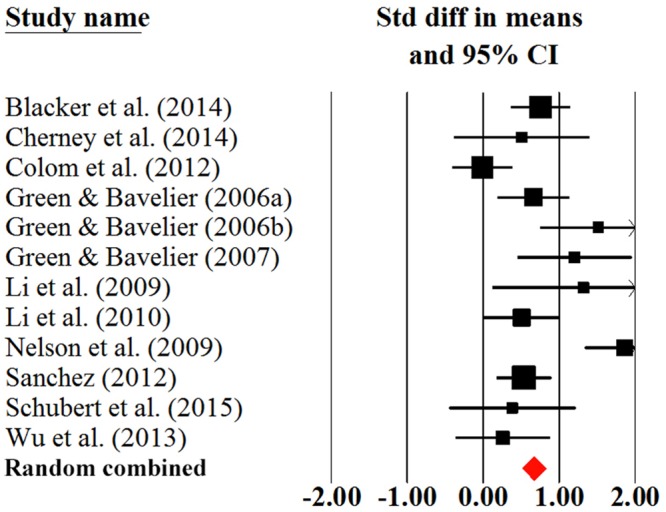
**Overall efficacy of action video game training on all cognitive outcomes in young adults**.

**FIGURE 4:**
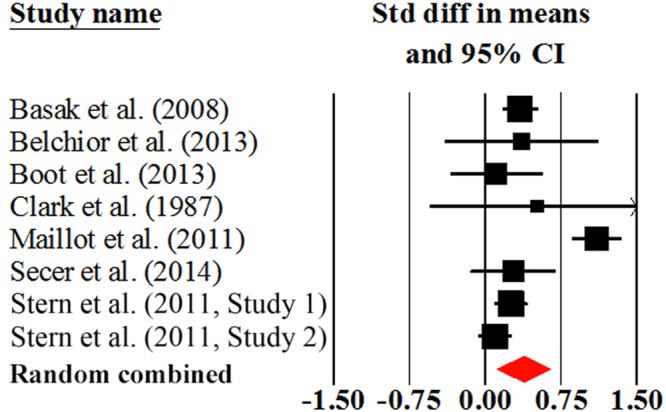
**Overall efficacy of action video game training on all cognitive outcomes in older adults**.

We calculated the AVG training effect on young and older adults, respectively. As shown in **Table 2**, for young adults, the magnitude for overall cognition was moderate (effect size = 0.75; **Figure 3**), the homogeneity test was significant across studies (*Q* = 42.27, *P* < 0.001). Relatively, the magnitude of overall cognition for older adults was small (effect size = 0.38; **Figure 4**), suggesting that older adults experienced a small benefit in cognition from AVG training. The homogeneity statistic also showed significant heterogeneity across the studies (*Q* = 48.14, *P* < 0.001). Egger’s tests revealed that the publication bias in overall cognition was not significant in both young and older adults (**Table 2**). The *Q*-test demonstrated that the magnitude of the effect size in young adults was significantly higher than that in older adults in overall cognition (*Q* = 13.17, *P* < 0.001).

We further analyzed the training effects on young and older adults for specific cognitive domains. Young adults benefited most in processing speed/attention (Cohen’s *d* = 0.81), followed by visuospatial ability (Cohen’s *d* = 0.70), and executive function (Cohen’s *d* = 0.64). These three effect sizes reached large or moderate magnitudes. We did not compute the effect size of memory in young adults due to a lack of data. Older adults also benefited from AVG training in executive function (Cohen’s *d* = 0.40), processing speed/attention (Cohen’s *d* = 0.37), memory (Cohen’s *d* = 0.33), and visuospatial ability (Cohen’s *d* = 0.29). The magnitudes for all these four domains were small. The young adults generally obtained higher effect sizes than older adults in each specific cognitive domain; the difference was significant in processing speed/attention (*Q* = 7.28, *P* = 0.007), but it was insignificant in visuospatial ability (*Q* = 2.77, *P* = 0.096) and executive function (*Q* = 0.09 and *P* = 0.762). Egger’s tests demonstrated that the publication bias was insignificant in specific cognitive domains in both young and older adults (**Table 2**). Due to the small number of studies contributed to specific cognitive domains in young and older adults, we should treat these results cautiously.

### Potential Moderators in Meta-Analysis of Overall Adults

The heterogeneity test in the overall meta-analysis showed that there was an obvious variance across different studies. Therefore, we investigated whether some potential moderator variables influence the AVG training effects. The potential moderator variables included the training session number, session length, total training duration, study quality, and types of control groups. We also investigated whether age, education, or the gender ratio of participants in the training group would influence the training effect.

The meta-regression analyses showed that the session number (Slop = -0.01, *Q* = 11.65, *P* < 0.001), duration of each session (Slop = -0.14, *Q* = 6.28, *P* = 0.012), total training duration (Slop = -0.01, *Q* = 15.65, *P* < 0.001), and age and education of the participants in the training group were negatively correlated with effect sizes (**Table 3**). The gender ratio of the training group was not significantly correlated with effect sizes (Slop = 0.11, *Q* = 0.10, *P* = 0.751; **Table 3**). The included studies demonstrated relatively high quality, ranging from six to nine points. The meta-regression analyses showed that the study quality was negatively correlated with effect sizes of AVG training, approaching significance (Slop = -0.08, *Q* = 3.39, *P* = 0.066). We conducted meta-analyses for the different control types and then compared effect sizes with the *Q*-test. The results showed that the mean effect sizes for the active-control group (Cohen’s *d* = 0.81, 95% CI was between 0.64 and 0.98) were significantly higher than those in the passive-control group (Cohen’s *d* = 0.33, 95% CI was between 0.24 and 0.41), *Q* = 24.60, *P* < 0.001 (**Table 3**). Among the 10 studies classified as passive-control studies, eight studies focused on older adults and two on young participants. In another 10 studies classified as active-control studies, all were focused on young adults.

**Table 3:** Analyses of potential moderators of effect sizes in overall adults.

Moderator variables	No of studies	Mean ES (SE)	95% CI	Slop	*Q*	*P*
Session number	12	0.53 (0.14)	0.26–0.81	-0.01	11.65	<0.001
Session length	15	0.56 (0.12)	0.32–0.80	-0.14	6.28	0.012^∗^
Total training duration	20	0.58 (0.10)	0.37–0.78	-0.01	15.65	<0.001
Age	18	0.60 (0.11)	0.38–0.82	-0.01	9.36	0.002^∗∗^
Male’s ratio	16	0.39 (0.08)	0.23–0.54	0.11	0.10	0.751
Education	6	0.43 (0.16)	0.11–0.74	-0.19	44.85	<0.001
Study quality	20	0.58 (0.10)	0.37–0.78	-0.08	3.39	0.066
Active-control type	10	0.81 (0.81)	0.64–0.98	NA		
					24.60	<0.001
Passive-control type	10	0.33 (0.04)	0.24–0.41	NA		

*Note: ^∗^*P* < 0.05; ^∗∗^*P* < 0.01. CI, confidence interval; ES, effect size; SE, stander error.*

## Discussion

In the present study, we used the meta-analysis approach to examine the cognitive efficacy of AVG training on healthy adults. The results demonstrated that healthy adults achieved moderate improvements in overall cognitive ability through AVG training. For specific cognitive domains, healthy adults achieved moderate benefits on visuospatial ability and processing speed/attention, nearly close to moderate benefits on executive function, and small benefits on memory. Young adults benefited more in overall and specific cognitive domains than older adults from the AVG training.

### The Overall Meta-Analysis of AVG Training in Overall Adults

In the present study, we found that AVG training enhanced both overall and specific cognitive functions in healthy adults. The extent of the overall efficacy was moderate, which was similar to recent meta-analytic findings on video game training (Powers et al., 2013). Powers et al. (2013) focused on the information processing domains (auditory processing, visual processing, executive functions, motor skills, and spatial imagery) and found that the overall effect size was 0.48, which approaches moderate magnitude. In the current meta-analyses, we focused on AVG training and different cognitive domains and replicated the positive training effects in the overall cognition in healthy adults.

While a host of studies have proven that there are positive effects of AVG training on cognition, the accuracy of its mechanism is still not well understood. During AVG training, due to the multimodal involvement of resources, game players may enhance the processing components with more widespread consequences (Hertzog et al., 2008). Therefore, AVG training has always been associated with broad enhancements in cognitive function due to its requirements of rapid and accurate reaction, switches between high concentrate attention and divided attention and other characteristics (Green and Seitz, 2015). The learning to learn theory was proposed to explain the broad transfer effect of AVG training (Bavelier et al., 2012b; Green and Bavelier, 2012). This theory posited that AVG training may improve general top-down controlling and probabilistic inference abilities, and game players would be guided to perform different tasks and experience a broad range of cognitive enhancements. Recent event-related potentials (ERPs) and neuroimaging studies demonstrated that AVG training facilitated brain plasticity in adults. Researchers found that AVG playing changed the cortical networks for complex visuomotor transformation (Granek et al., 2010), efficient attentional resource allocation in fronto-parietal network (Bavelier et al., 2012a), enhanced functional connectivity between attentional and sensorimotor networks (Gong et al., 2015), and increased amplitudes in subsequent visual ERPs (Wu et al., 2012). Specifically, in older populations, Anguera et al. (2013) revealed that the game-induced increase of midline frontal theta power was correlated with the preservation of multitasking performance and the transfer task. The structural imaging studies also found increased gray matter volume in the right posterior parietal (Tanaka et al., 2013) and insula (Gong et al., 2015) in adults. The dorsal striatum volume was found to be associated with video game learning improvement (Erickson et al., 2010). These results suggest that the brain structural and functional changes may facilitate the cognitive flexibility and transfer to the untrained tasks. Future studies are warranted to investigate the underlying transfer mechanisms of AVG training.

The current analyses suggested that healthy adults achieved moderate benefits from AVG training in processing speed/attention, which was in line with previous findings. Researchers found that the AVG players experienced enhancement in various aspects of attention, such as sustained attention, visual selective attention, and divided attention (Greenfield et al., 1994; Green and Bavelier, 2003; Feng et al., 2007). While playing the AVG, players may experience enhancements in sustained attention and general concentration (Mayas et al., 2014), as well as attention resources (Dye et al., 2009); therefore, the players devote more processing resources to the tasks and optimize the processing efficiency of the tasks. The AVG usually requires players to process information quickly and make quick responses, which determines success or failure in the games. Under such requirements, researchers found that players learned to make full use of the informative cues (Karle et al., 2010) and experienced an increase in the spatial resolution of visual processing across the visual field (Green and Bavelier, 2007). These experiences may result in the increase of attention and processing speed.

We observed a moderate effect size in visuospatial ability in the overall meta-analyses. Previous studies suggested that healthy adults’ visuospatial ability had significant plasticity, which can be enhanced through video game playing (Dorval and Pepin, 1986; De Lisi and Wolford, 2002; Ferguson, 2007), spatial task practice (Wright et al., 2008), or organized athletic activities (Ginn and Pickens, 2005). All of these experiences have the same requirements of manipulating and using visuospatial information repeatedly in a goal-directed manner. The AVG usually requires players to perceive the relationship between multiple objects in the visuospatial environment, which may lead to transfer effects and enhanced visuospatial processing in healthy adults (Basak et al., 2008).

We found that healthy adults experienced a small moderate training effect in executive function in the studies. Toril et al. (2014) found a negligible effect size of executive function in older adults. The differences between these two meta-analyses may be because Toril et al. (2014) exclusively focused on older adults, whereas we included both young and older adults. We found that older adults benefited less than young adults from the AVG training. Different video game types may also result in inconsistent results. Toril et al. (2014) investigated the training effects of all types of video games, whereas we were interested in the AVG type exclusively. Although the AVG is one type of video game, they are still different from common video games to some degree and contain their own features. The AVG often includes unpredictability, quick presentation, and response requirements, high perceptual load, selections between multiple action plans and an emphasis on peripheral processing (Hubert-Wallander et al., 2011; Oei and Patterson, 2013). These characteristics lead to greater plastic ability in cognitive control. Moreover, Green et al. (2012) claimed that compared to other types of video game, the AVG may be more beneficial in information processing, which included executive function.

We observed a small effect on memory in the overall meta-analysis. Because only three studies with older participants contributed to the memory domain, the results may be unreliable, and we should be cautious with this result. However, this conclusion is in line with prior findings that the memory of older adults remains plastic and may be promoted through relevant cognitive training (e.g., Neely and Backman, 1995; Calero and Navarro, 2007; Cheng et al., 2012; Karbach and Verhaeghen, 2014; Park et al., 2014; Shah et al., 2014; Ballesteros et al., 2015). Considering the requirements of the information maintenance in the AVG, AVG training indeed has the potential to enhance the memory of adults. More studies are warranted to investigate whether AVG training can enhance the memory of healthy adults.

### The AVG Training Meta-Analyses in Young and Older Adults

We further examined the AVG training effects on young and older adults and found that AVG training has a positive effect on both overall and specific cognitive domains. The results demonstrated that young adults benefited more than older adults in overall cognition and in specific domains. Effect sizes varied from moderate to large magnitudes in young adults but were all within a small range for older adults.

The categories of cognitive training were classified into strategy-based training, processing-based training, and multi-domain training (Noack et al., 2009; Karbach and Verhaeghen, 2014). Strategy-based training often resulted in amplification effects, showing that young adults achieve more gains than older adults, whereas the process-based training leads to compensation effects, showing that older adults benefit more than young adults. The amplification model shows that young adults have more efficient cognitive resources to acquire and implement new strategies; therefore, they achieve more gains than older adults (Verhaeghen and Marcoen, 1996). The compensation model suggests that young adults already experienced optimal performance and leaves less room for improvement (Karbach and Verhaeghen, 2014). AVG training is considered multi-domain training; however, the underlying mechanism of the training effects is unclear. The above classification and findings (Noack et al., 2009; Karbach and Verhaeghen, 2014) offer insights to explain the current findings. In the current meta-analyses, we found that young adults achieved more gains than older adults. The meta-regression analyses also showed that the greater the age of participants in AVG training is, the lower the gains they achieved. These findings provide evidence for the amplification model, and these effects, induced by the multi-domain AVG training, are similar to strategy-based training. Previous studies demonstrated that the short-term and long-term AVG training induce strategy changes (Nelson and Strachan, 2009; Clark et al., 2011). Clark et al. (2011) reported that experienced gamers possessed better top-down search strategies and performed better in a change detection tasks. Moreover, some AVGs are real-time strategy games (for example, Rise of Nations), which are more reliant on sustained attention, updating, and planning and promote executive control processing (Latham et al., 2013).

However, the meta-regression analyses for years of education yielded different patterns and supported the compensation model. Calero and Navarro (2007) noted that the level of education mediated the post-training improvement. Education was found to exert a positive influence on memory training in older adults (West et al., 1992; Inouye et al., 1993). The present meta-regression analyses find that education is negatively correlated with the effects of AVG training. To our knowledge, few studies have investigated the effect of education on video games. We speculate that this is because people with more years of education already have optimal performance and have less room to improve, whereas participants with fewer years of education have lower baseline scores and may benefit more from training. This result supports the compensation model. It should be noted that the observed compensation effect is not age-related. In total, the current meta-analytic findings demonstrate that young adults achieve more benefits than older adults during AVG training, which supports the amplification model, whereas the demographic variables (age and years of education) yielded different patterns. These results imply that these different theoretical models may be integrated and cooperatively enhance adults’ cognitive abilities during AVG training.

Previous behavioral studies present consistent findings that young adults maintain more plasticity than older adults (e.g., Pascual-Leone et al., 2005; Bialystok, 2006). Neuroimaging studies show that young adults may have greater neural plasticity than older adults. Nyberg et al. (2003) found that both young and older adults experienced enhancement in their memory performance after receiving visuospatial memory training, whereas only young adults presented increased activity in the occipito-parietal and frontal regions. Dahlin et al. (2008) found transfer effects in young adults – but not in older adults – in computer-based executive function training; they concluded that the age-related deficits in striatal functioning might result in limited transfer effects in older adults. Wenger et al. (2012) reported that both young and older adults benefited from spatial navigation training in the behavioral performance, whereas training-related cortical thickening in the precuneus and paracentral lobule was only found in young adults. Maass et al. (2015) further suggested that the preserved capacity of the hippocampus, which was important for functionally relevant plasticity, decreased with increasing age. The aforementioned evidence suggests that young adults have relatively higher cognitive function and potentially greater cognitive and neural plasticity than older adults.

In summary, although the training effect differs in magnitude, AVG training indeed has a positive influence on both young and older adults, confirming the presence of cognitive plasticity throughout adulthood and supporting the role of the AVG in serving as an effective tool for cognitive improvement in both young and older adults.

### The Moderator Variable Analyses in the Overall Meta-Analysis

The moderator variable analyses suggest that the cognitive effects of AVG training were modulated by various moderators. The session duration, session number, and the total training duration were significantly negatively correlated with AVG training effect sizes. These results are consistent with previous meta-analytic findings (Powers et al., 2013; Lampit et al., 2014; Toril et al., 2014). The *temporal discounting hypothesis* (Green et al., 1994) showed that future rewards are less valuable than immediate rewards. The longer the time that participants spend on training, the more they may feel that they will pay more and receive fewer benefits, so the motivation to continue playing the game decreases and leads to less training efficacy. In the AVG training group, the participants played games for an average of 22.31 h (ranging from 16.00 to 32.99). We speculated that motivation decline during the long period of training may lead to decreased performance. After carefully checking the included studies and training data, we found that most of the studies reported continuous improvement in the game performance through the training process. Therefore, the motivation decline inference did not explain this finding. According to previous studies, the perceived flow of effects such as arousal, engagement, reward, feedback, and enjoyment may influence the training effect with time (Csikszentmihalyi, 2008; Belchior et al., 2012); however, these factors are difficult to systematically assess in a meta-analytic study, and the potential reasons need further investigation. The meta-regression analysis with regard to age and years of education are discussed above.

Previous cognitive training studies have highlighted the modulatory effect of control group types, declaring that studies with passive-control obtain more favorable effects than those with active-control (Melby-Lervag and Hulme, 2013; Danielsson et al., 2015). Toril et al. (2014) conducted a meta-analysis on video game training and found similar effects. The placebo effect may contribute to the differences in types of control group. Boot et al. (2011) claimed that participants would have an expectation in the experimental and active-control groups but not in the passive-control group. If the training methods in the experimental group and active-control group are similar, the training effect may be underestimated in the study, leading to a lower effect size in the active-control studies than in the passive-control studies (Huntley et al., 2015). Surprisingly, an opposite effect of control type is found in our meta-analysis; the efficacy of AVG training in active-control groups is greater than studies with passive-control groups. Given that all of the 10 active-control studies focused on young adults, whereas 8 of the 10 passive-control studies are on older adults, as discussed previously, young adults obtain greater efficacy from the training than older adults, there may be mixed effects of age and control group type in the present meta-analysis. To understand more clearly the influences of control types in AVG training, future studies are needed to continue to investigate this question.

### Limitations

As a quantitative meta-analyses about the AVG training effect on young and older adults, to obtain reliable and reproducible results, we employed strict inclusion and exclusion criteria and computed the effect sizes of overall cognition as well as specific cognitive domains. However, there are still some limitations in the present analyses. First, although we searched and scrutinized the published references carefully, the number of included studies was still not large. The main reason was that most of the previous studies were cross-sectional, and only a small number of studies focused on AVG training. Furthermore, our inclusion and exclusion criteria were stringent: the included AVG training studies must have a control group, both the AVG training and the control group should provide a pre- and post-test of the same cognitive outcomes or amenable data to calculate the treatment effect for the meta-analyses, which excluded many potential studies. Second, the sample size of the included studies was small, and the included studies did not report the power and sample size estimation. Third, the present meta-analyses lacked studies that targeted middle-aged adults because few studies have examined the AVG training effects in this age group. Future studies aiming to investigate the AVG training effect of middle-aged adults are welcome to fill the gap in this research domain. Fourth, because few studies provided follow-up tests, we did not investigate the follow-up effect of AVG training. Finally, the current study focuses only on healthy adults, so we are unsure as to whether the results can be generalized to adolescents or clinical populations.

### Future Directions

The present findings provide several directions for future AVG training studies. Specialized and individualized AVG training can be designed to achieve certain training goals. To promote cognitive control in older adults, Anguera et al. (2013) combined the characteristics of multi-tasking and a driving game and designed a three-dimensional video game. They found that older adults in the training group experienced a reduction in multi-tasking costs in comparison with older adults in the passive and active-control groups. In future studies, according to the ages of participants and certain training goals (e.g., improving attention, visuospatial processing, and working memory), researchers may use self-designed AVG to achieve better training effects.

The AVG training may extend to the clinical populations or certain populations. We focused on healthy adults in the present meta-analysis; however, recent studies have found that video games may also be used as a treatment in clinical populations, such as stroke (Givon et al., 2015) or amblyopia (Li R.W. et al., 2011). Moreover, Green and Bavelier (2012) proposed that AVG might be used as a training method for jobs requiring precision, such as performing endoscopic surgery and piloting unmanned aerial drones.

The present meta-regression results have important implications for future AVG training studies. The current results found that AVG training studies with a passive-control group or an active-control group resulted in different effects. Klingberg (2010) proposed that including an active-control group should be the gold standard in training studies. Therefore, future AVG training studies are encouraged to include an active-control group. Moreover, the moderator variables results reveal that the demographic variables (age, education) and training regimen variables (session number, duration of each session, and total training duration) may influence the training effects. These findings may also provide references for future AVG training studies.

## Conclusion

To summarize, the present meta-analysis demonstrates that healthy adults benefit from the AVG training in both overall and specific cognitive domains. Young adults benefit more from the AVG training than older adults in both overall and in separate cognitive domains. Age, education, study quality, and some methodological factors, such as the session duration, session number, total training duration, and control group type, modulated these effects. The current findings demonstrate that AVG training can serve as an effective intervention tool for cognitive improvement in healthy adults, especially for young adults.

## Author Contributions

PW and H-JL conceived the idea and wrote the manuscript; PW, X-TZ and TM searched and coded the references; H-HL and X-NZ contributed towards writing the paper.

## Conflict of Interest Statement

The authors declare that the research was conducted in the absence of any commercial or financial relationships that could be construed as a potential conflict of interest.
